# Association of genetic polymorphisms with psychological symptoms in cancer: A systematic review

**DOI:** 10.1016/j.apjon.2021.11.006

**Published:** 2021-12-25

**Authors:** Sek Ying Chair, Bernard M.H. Law, Judy Y.W. Chan, Winnie K.W. So, Mary M.Y. Waye

**Affiliations:** a The Nethersole School of Nursing, Faculty of Medicine, The Chinese University of Hong Kong, Hong Kong SAR, China; b Asia-Pacific Genomic and Genetic Nursing Centre, The Nethersole School of Nursing, Faculty of Medicine, The Chinese University of Hong Kong, Hong Kong SAR, China; c The Croucher Laboratory for Human Genomics, The Nethersole School of Nursing, Faculty of Medicine, The Chinese University of Hong Kong, Hong Kong SAR, China

**Keywords:** Gene polymorphisms, Anxiety, Depression, Posttraumatic stress disorder, Cancer

## Abstract

Cancer patients suffer from a repertoire of symptoms, including such psychological and psychiatric symptoms as anxiety, depression, and posttraumatic stress. Exploration of genetic factors that modify the risk and severity of these symptoms may facilitate the development of personalised care plans for managing these symptoms. This review aims to provide an overview on the variations in genes that may contribute to the occurrence and severity of anxiety, depression, and posttraumatic stress disorder (PTSD) among cancer patients. Literature search was performed in nine English and Chinese electronic databases, and extracted data are presented narratively. The reporting quality of the included studies was assessed using selected items of The STrengthening the REporting of Genetic Association (STREGA) checklist. Twenty-nine studies were included in the review. Most studies involved breast cancer patients, while patients of other cancer types appeared to be understudied. A number of studies reported the association between genes involved in inflammatory pathways and depression and anxiety. Other genes found to show associations with anxiety, depression, and PTSD among cancer patients are those involved in neurotrophic signalling, serotonergic signalling, regulation of stress response, antioxidation, dopamine catabolism and cellular apoptosis, despite some inconsistencies in findings between studies. Our review highlighted a need for further research for enhancing our knowledge on the association between genetic variations and anxiety, depression, and PTSD of patients of various cancer types. Future studies examining such associations in patients of various cancers should utilise standardised instruments for outcome assessments and stratify the patients based on their age for analysis.

## Introduction

Cancer is currently one of the most common diseases worldwide, and it was estimated that more than 19 million new cancer cases were reported in 2020 worldwide.^1^ Despite the high prevalence of cancer, advancements in the development of cancer therapeutics over recent years had resulted in a reduction of mortality among cancer patients. Nevertheless, the improved survival of cancer patients had brought about the need for further resources for oncology nursing care of the increased number of cancer survivors, pertaining to the management of cancer symptoms and adverse effects caused by cancer treatment. Indeed, these symptoms were known to exhibit a detrimental effect to the quality of life of the patients, including those having completed cancer treatment.^2^, ^3^, ^4^ In view of this, strategies need to be developed for the effective management of cancer-related symptoms among patients, notably through a personalised approach in oncology care which could help improve treatment outcomes and reduce its adverse effects.^5^^,^^6^

Among the cancer-associated symptoms, anxiety and depression are two of the most common that are faced by cancer patients, and they are potentially more prevalent among older and female patients.^7^ In addition to their high prevalence, these psychological symptoms were reported to be occurring at a high severity level in cancer patients,^8^ and they could persist even patients have completed cancer treatment.^9^ Indeed, these symptoms were reported to be prevalent particularly among breast cancer patients having completed treatment, where their prevalence ranges between 9% and 66%, as reported by various studies.^10^ These psychological symptoms are contributed by the physical symptom burden experienced by cancer patients as a result of cancer treatment,^11^^,^^12^ together with financial problems and lack of social support.^13^^,^^14^ Moreover, the currently ongoing coronavirus disease 2019 (COVID-19) pandemic could play a role in exacerbating the development of these psychological symptoms among cancer patients. They may feel anxious about their disease status owing to the potential interference of their treatment course during the pandemic, when healthcare providers become overwhelmed by the increasing number of COVID-19 patients.^15^ Cancer patients may also feel depressed for the need to practice social distancing during the pandemic, which leads to their feeling of loneliness and being socially isolated.^15^ These symptoms would exhibit a number of detrimental effects to both the well-being and clinical outcomes of cancer patients, including decreased treatment adherence,^16^, ^17^, ^18^ increased cancer mortality,^19^ and impairment of patients' quality of life.^20^, ^21^, ^22^

Moreover, cancer survivors could also experience posttraumatic stress from the traumatic experience of being diagnosed and treated for cancer, leading to the development of posttraumatic stress disorder (PTSD).^23^ A previous meta-analysis indicated that PTSD could occur in about 10% of adult cancer patients who had completed treatment.^24^ Notably, anxiety and depressive symptoms were found in multiple studies to be associated with posttraumatic stress among cancer patients,^25^, ^26^, ^27^, ^28^ suggesting an inter-relationship between these psychological conditions and that cancer survivors are in high need of interventions that concurrently address these three conditions. In view of the high prevalence of these related psychological and psychiatric symptoms and their negative impact on patients, the exploration of effective care strategies that address these symptoms among cancer patients is of paramount importance.

With the growing importance of personalised care in oncology, research has been directed towards the exploration of the genetic factors that would affect the occurrence and severity of cancer-related symptoms among patients, including psychological/psychiatric symptoms such as anxiety, depression, and posttraumatic stress. One of these widely studied genetic factors is the variations in the nucleotide sequence in genes or single nucleotide polymorphisms (SNP). Reportedly, SNPs in various genes were shown to be associated with the risk of various diseases such as cancer,^29^ heart diseases,^30^ and neurodegenerative diseases.^31^ Moreover, SNPs were demonstrated to be associated with various symptoms or symptom clusters reported among cancer patients,^32^, ^33^, ^34^ leading to modifications in the severity level of these symptoms. Further research of such association may facilitate the exploration of markers that may be used to identify individuals at higher risk of experiencing more severe symptoms. Furthermore, with the identification of genes that are associated with the occurrence and severity of symptoms, the molecular pathways that may contribute to the development of these symptoms can also be identified.

Previously, two reviews on the association of SNPs with certain psychological symptoms among cancer patients were published.^35^^,^^36^ Nevertheless, these reviews were limited to the exploration of such association among breast cancer patients. A review that comprehensively explore the genetic polymorphisms associated with the aforementioned psychological/psychiatric symptoms is needed, which helps open up further avenues of research into the genes that can be targeted for addressing the aforementioned psychological symptoms among cancer patients. The aim of this review is to provide an overview of the variations in genes that may contribute to the occurrence and severity of anxiety, depression, and posttraumatic stress among cancer patients either undergoing or have completed treatment.

## Methods

### Search strategy

We conducted a literature search in PubMed, PsycINFO, CINAHL, OVID MEDLINE, and Web of Science in July 2021, to retrieve articles that meet the eligibility criteria of the review. A further literature search was also conducted in July 2021 in Chinese databases including Wanfang, CNKI, CQVIP, and SinoMed to retrieve relevant studies conducted in Mainland China. Moreover, the reference section of the studies deemed eligible for inclusion was screened to identify any further relevant studies for inclusion. The search strategies employed for literature search in the English and Chinese databases are presented in Supplementary Tables S1 and S2, respectively.

### Eligibility criteria

To be eligible for inclusion in the review, the articles need to be reporting observational studies involving cohorts of patients with cancer of any type, at any stage of their cancer, either receiving or have completed cancer treatment. These studies should also be reporting the association of SNPs or genetic variants with psychological outcomes limited to depression, anxiety, and post-traumatic stress in the patients. Studies reporting such association with symptom clusters of psychological outcomes and other cancer symptoms were excluded. All studies reporting animal studies or studies involving the use of cell lines or primary cells were excluded. Articles that were not reporting original studies were also excluded. Further, studies with samples of patients having benign tumours were excluded.

To determine whether the retrieved articles were eligible for inclusion, the titles and abstracts were screened by two authors independently. The software tool Covidence was used during the screening process. Those considered relevant to the review aims were selected for further review by examining the full-text of the articles. Disagreements on whether articles should be included or excluded were resolved by discussion between the two authors.

### Data extraction

Data extracted from the included studies include sample size, characteristics of the sample, type of cancer examined, the name of SNP examined and its rs number if provided, name of the gene that the SNP is located, psychological symptoms of patients explored and the instruments used for assessing such symptoms, and the major findings pertaining to the association between the tested SNPs and anxiety, depression, and posttraumatic stress among subjects. Data were presented narratively and in a tabular format. Data extraction was first conducted by a reviewer, and the accuracy of the extracted data was verified by a second reviewer. Any disagreements in the extracted data were resolved by discussions.

### Quality assessment of included studies

The quality of the included studies was assessed using the selected nine items from the STrengthening the REporting of Genetic Association (STREGA) checklist, as conducted in previous reviews.^35^^,^^36^ The checklist was first developed by Little et al.,^37^ and was used as a guideline to enhance the comprehensiveness of the reporting of genetic association studies. In the quality assessment, the studies were assessed on the reporting of six major areas, including (1) genotyping methodologies, the location where it was performed and whether the genotyping was done in batches, (2) outcomes of genotyping, including error rate and call rate, and the number of samples that were successfully genotyped, (3) methodologies in controlling for confounders such as population stratification, (4) how genotypes or haplotypes were inferred, (5) whether the Hardy–Weinberg equilibrium was considered, and (6) whether the study was a replicated effort of previous studies. One mark was given to each included study for each item that was reported, and the marks were summed to give a total score for each study. The quality assessment was conducted by two authors independently, and disagreements in the ratings were resolved by discussion.

## Results

### Search results

**Figure 1:**
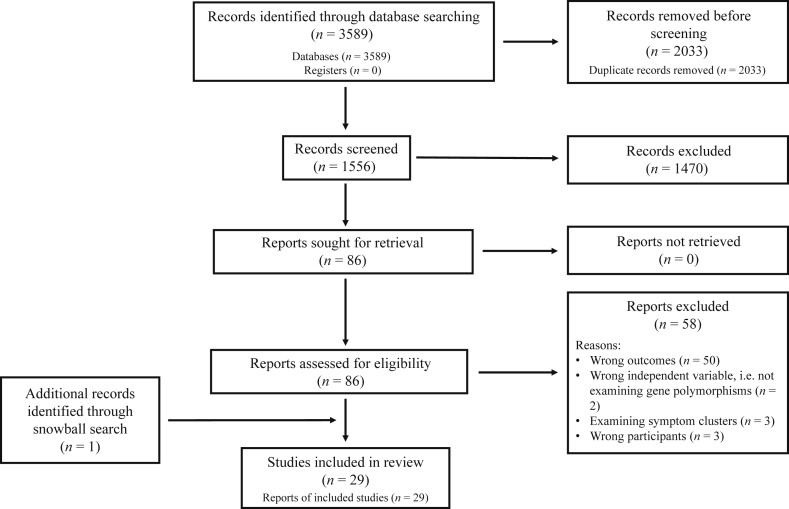
The PRISMA diagram.

A literature search using the aforementioned nine electronic databases yielded a total of 3589 articles, of which 2033 were duplicates. After their removal, the titles and abstracts of the remaining 1556 articles were screened, and 1470 articles were excluded at this stage for their lack of relevance to the objectives of this review or being studies involving animals, cell lines or primary cells. The full-text of the remaining 86 articles were read and a further 58 articles were excluded for measuring outcomes irrelevant to the aim of this review (*n* = 50), having a sample with irrelevant populations (*n* = 3), not reporting the effect of gene polymorphisms on the desired outcomes (*n* = 2) and assessing symptom clusters rather than individual symptoms of anxiety, depression or posttraumatic stress (*n* = 3). A snowball search by screening the reference list of the included studies yielded one further study for inclusion. Twenty-nine studies were therefore included in this review. The PRISMA diagram depicting the flow of the literature search is shown in Figure 1.

### Results of quality assessment

The quality ratings of each included study are presented in Table 1. Overall, the quality of reporting genetic associations among the studies was moderate, with quality scores ranging between 11% and 78%. All the studies reported the methodology of genotyping appropriately, although two studies did not provide sufficient details of the methodologies used.^38^^,^^39^ Most studies reported the number of samples that were successfully genotyped (83%), how the issue of population stratification was addressed (76%) and how genotypes and haplotypes were inferred (69%). Most (76%) had also considered the Hardy–Weinberg equilibrium in their analysis of the genetic associations. Nevertheless, only a small proportion of the included studies reported issues including error rate and call rate (14%), the location of laboratory where genotyping was performed (14%) and whether genotyping was performed in batches (3%). Finally, 45% of the included studies indicated that the study was the first to show the association of gene polymorphisms of interest with the examined psychological symptoms. An additional study was a replicative effort of a previous study.^38^ The remaining studies did not make indications on whether their studies were replicative to previous studies.

**Table 1:** Critical appraisal of included studies.

Study (Reference)	Item 1	Item 2	Item 3	Item 4	Item 5	Item 6	Item 7	Item 8	Item 9	Score (% score)
Bower et al., 2013^40^	Yes	No	No	No	Yes	Yes	No	Yes	No	4 (44%)
Brackett et al., 2012^41^	Yes	Yes	No	No	Yes	No	Yes	No	No	4 (44%)
Chen et al., 2019^51^	Yes	Yes	No	No	Yes	Yes	Yes	Yes	No	6 (67%)
Cihan et al., 2017^66^	Yes	No	No	No	Yes	Yes	Yes	Yes	Yes	6 (67%)
Dai et al., 2008^55^	Yes	No	No	No	No	Yes	No	Yes	Yes	4 (44%)
Dooley et al., 2017^42^	Yes	No	No	Yes	Yes	Yes	No	Yes	No	5 (56%)
Eberhard et al., 2010^39^	No	No	No	No	Yes	No	No	No	No	1 (11%)
Feng et al., 2020^43^	Yes	No	No	No	Yes	Yes	No	Yes	Yes	5 (56%)
Gilbert et al., 2012^44^	Yes	No	No	No	Yes	No	Yes	No	No	3 (33%)
Grassi et al., 2010^61^	Yes	No	No	No	Yes	Yes	No	Yes	No	4 (44%)
Guo et al., 2019^52^	Yes	No	Yes	No	Yes	Yes	Yes	Yes	No	6 (67%)
Kang et al., 2012^56^	Yes	No	No	No	Yes	Yes	Yes	Yes	No	5 (56%)
Kim et al., 2012^57^	Yes	No	No	No	Yes	No	No	No	Yes	3 (33%)
Kim et al., 2012^58^	Yes	No	No	No	Yes	Yes	Yes	Yes	Yes	6 (67%)
Kim et al., 2013^59^	Yes	No	No	No	Yes	Yes	No	Yes	Yes	5 (56%)
Kim et al., 2018^45^	Yes	No	Yes	No	Yes	Yes	No	Yes	Yes	6 (67%)
Koh et al., 2014^60^	Yes	No	No	No	Yes	Yes	Yes	Yes	No	5 (56%)
Lou et al., 2021^53^	Yes	No	No	No	Yes	Yes	Yes	Yes	Yes	6 (67%)
Luo et al., 2020^54^	Yes	Yes	Yes	No	Yes	Yes	Yes	Yes	No	7 (78%)
Miaskowski et al., 2016^46^	Yes	No	No	No	No	Yes	Yes	Yes	Yes	5 (56%)
Reyes-Gibby et al., 2013^47^	Yes	No	No	No	No	No	Yes	No	Yes	3 (33%)
Saad et al., 2014^48^	Yes	No	No	No	No	Yes	Yes	Yes	Yes	5 (56%)
Schillani et al., 2012^62^	Yes	No	No	No	Yes	Yes	Yes	Yes	No	5 (56%)
Sharpley et al., 2018^65^	Yes	No	No	No	Yes	No	Yes	No	No	3 (33%)
Suppli et al., 2015^64^	Yes	No	No	No	Yes	Yes	Yes	Yes	No	5 (56%)
Suppli et al., 2017^38^	No	No	No	No	No	Yes	Yes	Yes	Yes	4 (44%)
Wang et al., 2019^49^	Yes	No	No	No	Yes	No	Yes	No	No	3 (33%)
Young et al., 2017^50^	Yes	Yes	No	No	Yes	Yes	Yes	Yes	No	6 (67%)
Zerbinati et al., 2021^63^	Yes	No	Yes	No	Yes	Yes	Yes	Yes	Yes	7 (78%)

Description of the assessment items. Item 1: Description of laboratory methods, including source and storage of DNA, genotyping methods and platforms; Item 2: Report on the error rate and call rate; Item 3: Indication on the centre at which the genotyping was performed; Item 4: Report on whether the genotyping was done in one single batch or a few smaller batches; Item 5: Report on the number of individual participants' samples were genotyped and how many of these samples were successfully genotyped; Item 6: Description on how to assess the level of and/or control for population stratification; Item 7: Description on any methods on determining (inferring) genotypes or haplotypes; Item 8: Statement on whether the Hardy–Weinberg equilibrium is considered; Item 9: Statement on whether this is the first report to report such genetic association or was it a replicated effort of a previous study, or both.

### Characteristics of included studies

The included studies were published between 2008 and 2021. The majority of them were conducted in the United States (*n* ​= ​11).^40^, ^41^, ^42^, ^43^, ^44^, ^45^, ^46^, ^47^, ^48^, ^49^, ^50^ The remaining studies were conducted in either China (*n* ​= ​5),^51^, ^52^, ^53^, ^54^, ^55^ South Korea (*n* ​= ​5),^56^, ^57^, ^58^, ^59^, ^60^ Italy (*n* ​= ​3),^61^, ^62^, ^63^ Denmark (*n* ​= ​2),^38^^,^^64^ Australia (*n* ​= ​1),^65^ Sweden (*n* ​= ​1),^39^ and Turkey (*n* ​= ​1).^66^ The sample size of these studies ranges between 33 and 7320. Most (48%; *n* ​= ​14) of them involved samples of breast cancer patients,^40^^,^^42^^,^^45^^,^^46^^,^^48^, ^49^, ^50^^,^^55^^,^^57^, ^58^, ^59^^,^^61^, ^62^, ^63^ three involved patients of gastric cancer,^53^^,^^56^^,^^60^ and two with liver cancer patients.^52^^,^^54^ Other studies involved samples of patients with blood cancer,^66^ brain cancer,^41^ colorectal cancer,^64^ head and neck cancer,^44^ lung cancer,^47^ prostate cancer,^65^ testicular cancer,^39^ or thyroid cancer.^51^ Two additional studies utilised samples where patients with different cancer types were mixed together.^38^^,^^43^ Of note, two included studies involved samples of paediatric cancer patients.^41^^,^^66^ Among the included studies, most examined the SNPs in the serotonin-transporter-linked promoter region (5-HTTLPR) (*n* ​= ​11), brain-derived neurotrophic factor (BDNF) (*n* ​= ​7) or various types of cytokines (*n* ​= ​5). A summary of the characteristics of the included studies is presented in Supplementary Table S3.

### Identified gene polymorphisms associated with anxiety, depression, and posttraumatic stress among cancer patients

#### Anxiety

Nine studies examined the gene polymorphisms associated with anxiety among patients with various cancer types.^39^^,^^41^^,^^46^^,^^50^^,^^53^^,^^56^^,^^60^^,^^62^^,^^65^

Three of these studies involved samples comprising breast cancer patients.^46^^,^^50^^,^^62^ Among these patients, two SNPs (rs1799964 and rs3093662) were identified in the tumour necrosis factor-alpha (TNF-α) gene showing association with their anxiety levels.^46^ Interestingly, the presence of the rare allele of these SNPs would confer a differential effect on the anxiety levels among these patients, suggesting that these SNPs may have different effects on the functions of TNF-α. In another study,^50^ the rs4680 polymorphism in the catechol-O-methyl transferase (COMT) gene was also shown to increase anxiety levels in these patients. Nevertheless, the gene polymorphism resulting in a deletion in 5-HTTLPR that leads to the expression of a shorter version of the serotonin transporter gene (referred to as “deletion SNP” hereafter) did not have any effect on anxiety levels among breast cancer patients,^62^ although it did lead to higher anxious preoccupation levels among patients in the longer-term. Carriers of the rare S allele were found to have a slower decline of anxious preoccupation scores over time compared with patients having the wild type L allele.

A further three studies involved samples with gastric cancer patients.^53^^,^^56^^,^^60^ The examined SNPs in the Bcl-2/adenovirus E1B 19 ​kDa-interacting protein 3 (BNIP3) and death-associated protein kinase 1 (DAPK1) genes were demonstrated to have no effect on the anxiety levels of these patients.^53^ In contrast, the three examined SNPs in the FK506 binding protein 5 (FKBP5–rs1360780; rs9296158; rs9470080), a gene implicated in stress response,^67^ appeared to have a genotype by time interaction effect on anxiety levels among patients.^56^ While patients bearing the homozygous wild-type allele for these three SNPs reported decreased anxiety levels during this longitudinal study, carriers of the rare allele of these SNPs exhibited increased anxiety across time. Likewise, the level of anxious preoccupation among gastric cancer patients appeared higher among carriers of the rare Met allele of the SNP in BDNF (rs6265), suggesting an association between anxiety and this SNP in gastric cancer patients.^60^ Nevertheless, in another study with a sample of prostate cancer patients, the association between self-reported anxiety levels and rs6265 was not observed.^65^

The positive association between anxiety levels and gene polymorphisms was also reported in an additional study in patients of other cancer types, where Brackett et al. reported that the presence of a rare allele leading to gene deletion and lack of expression of the glutathione S-transferase Mu 1 (GSTM1) gene could result in higher anxiety levels in paediatric medulloblastoma patients.^41^

Eberhard et al.^39^ also examined the association between the polymorphism on the number of CAG and GGN repeats in the androgen receptor gene and anxiety of male testicular cancer patients. No such association was observed in their study, indicating that the length of the CAG and GGN repeats had no effect on modifying anxiety risk among the patients.

#### Depression

Twenty-two studies reported the gene polymorphisms that are associated with depression among various types of cancer patients, primarily breast, lung, colorectal, and gastric cancers.^38^^,^^40^, ^41^, ^42^, ^43^, ^44^, ^45^^,^^47^, ^48^, ^49^^,^^51^^,^^53^^,^^55^, ^56^, ^57^, ^58^, ^59^^,^^61^^,^^62^^,^^64^, ^65^, ^66^

##### Breast cancer

Eleven studies involved samples of breast cancer patients.^40^^,^^42^^,^^45^^,^^48^^,^^49^^,^^55^^,^^57^, ^58^, ^59^^,^^61^^,^^62^ A number of genes coding for pro-inflammatory cytokines were identified to have SNPs that could be associated with depression in these patients. For example, having a homozygous rare allele of the −511 ​C ​> ​T SNP of interleukin-1 beta (IL-1β) was associated with higher depression risk,^59^ while having that of the rs1800795 and rs2069840 of interleukin 6 (IL-6) could confer higher depression severity.^40^^,^^48^ Carriers of the rare allele of rs9376268 in the interferon-γ receptor 1 (IFNGR1) gene were also shown to have more severe depression.^48^ However, individuals having a homozygous rare allele for the rs1799964 SNP of TNF-α would have decreased depression severity, as evidenced by the lower odds of these individuals being in the subsyndromal class of depression.^48^ Interestingly, SNPs in the anti-inflammatory cytokines interleukin-4 (IL-4) and interleukin-10 (IL-10) were reported to have no association with depression in breast cancer patients.^59^ No such association was reported for SNPs in the pro-inflammatory interleukin-8 (IL-8) in breast cancer patients either.^59^

Contradictory findings were obtained from studies examining the association between depression and the deletion SNP of 5-HTTLPR among breast cancer patients. While two independent studies showed that having a homozygous rare allele leading to expression of the shorter version of serotonin transporter could lead to higher depression severity,^45^^,^^55^ the other studies demonstrated no effect of this SNP on depression risk and/or severity.^57^^,^^58^^,^^61^^,^^62^ In a longitudinal study, Wang et al. also showed that those having at least one copy of the rare allele exhibited an increase in depression while those with wild-type alleles did not.^49^ Nevertheless, Kim et al.^57^ indicated that the association between this deletion SNP and depression would be established if patients perceived a poor body image and sexual function, where carriers of the rare allele who had a poor body image would have greater depression severity. Regarding SNPs in other genes involved in the serotonergic pathway, Kim et al.^58^ reported that SNPs in the serotonin 2a receptor (5-HTR2a) and variations in the number of tandem repeats in the intron region of the serotonin transporter gene exhibited no effect on depression risk among breast cancer patients.

Finally, the rs6265 polymorphism of BDNF was found in two studies to be associated with depression among breast cancer patients. While Dooley et al.^42^ reported an elevated severity of depression contributed by increased C reactive protein production among carriers of the rare Met allele, Kim et al.^58^ demonstrated that having a homozygous genotype of the rare Met allele is associated with increased risk of prevalent depression and persistent depression.

##### Lung cancer

The association of SNPs in inflammatory cytokine genes with depression among lung cancer patients was examined by Reyes–Gibby et al.^47^ Most of the examined SNPs showed no associations with depression risk in these patients, including interleukin-1 alpha (IL-1α), tumour necrosis factor-beta (TNF-β) and the β subunit of IL-10 receptor. However, the −251 ​T ​> ​A polymorphism of IL-8 showed a marginally significant level of association with depression, where the possession of the rare allele conferred a lowered risk of severe depression.

##### Colorectal cancer

Suppli et al.^64^ examined the association of the deletion SNP and the rs25531 SNP of 5-HTTLPR with depression in colorectal cancer patients, based on the extent of anti-depressant use among these patients. Both SNPs were found not to be associated with depression risk nor severity among colorectal cancer patients.

##### Gastric cancer

Two independent studies examined the associations of SNPs in genes with depression among gastric cancer patients.^53^^,^^56^ Interestingly, Lou et al.^53^ demonstrated that the rs10781582 polymorphism in the BNIP3 gene is associated with depression in these patients, by showing that the possession of the rare allele would confer a reduced depression risk, making them less prone to this psychological symptom. Nevertheless, another examined BNIP3 SNP, rs3793742, exhibited no effect on patients' depression risks. Likewise, rs1329600 in DAPK1 is not associated with depression in these patients.

Kang et al.^56^ reported the association of two SNPs in FKBP5 (rs9296158 and rs9470080) with depression severity among gastric cancer patients. They demonstrated that both SNPs showed a genotype by time interaction effect with depression level in these patients. Patients having a copy of the rare allele in their genotype exhibited an increase in depression level over time, while the depression level among those having the wild type allele did not. The SNP rs9296158 was even shown to be a significant predictor of depression score among these patients. Another FKBP5 SNP, rs1360780, also showed a trend for a genotype by time interaction with depression severity of the patients (*P* ​= ​0.075), where the increase in depression severity over time among the homozygous wild type patients was reported to be smaller, compared to those having at least one copy of the rare allele. Of note, however, although genotype by time interaction effect was observed for both rs9296158 and rs9470080, where the extent of the change in depression severity was different among patients with various genotypes, no significant difference in depression severity was observed among these patients at baseline.

##### Other cancer types

Additional SNPs associated with depression among cancer patients were also revealed using patients with other cancer types. Brackett et al.^41^ showed that the SNP leading to the deletion of the GSTM1 gene could result in higher depression severity among paediatric medulloblastoma patients, demonstrating that this SNP is not only associated with anxiety, but also depression, in these patients. Among paediatric leukaemia patients, Cihan et al.^66^ showed that the BclI SNP in the nuclear Receptor Subfamily 3 Group C Member 1 (NR3C1) gene, a glucocorticoid receptor with roles in stress response, is associated with depression, where rare allele carriers were reported to have higher depression risks. The deletion SNP of 5-HTTLPR was also one of the candidates for such association, as significantly more papillary thyroid carcinoma patients with homozygous rare allele were reported to have depression, signifying the higher depression risk among these patients.^51^ Being the rare allele carrier of this SNP among head and neck cancer patients was also reported to exhibit increased odds of having depression, but the increase was not statistically significant.^44^ Finally, the rs6265 SNP of BDNF was also examined for its association with depression severity among prostate cancer patients,^65^ and a mixed sample of patients of different cancer types with 91% prostate cancer patients,^43^ but no effect was reported for this SNP on patients' depression severity in both studies.

In addition to colorectal cancer patients, Suppli et al.^38^ had also examined whether SNPs in genes are also associated with a mixed sample of patients with either colorectal, pancreas, lung, breast, prostate, corpus uteri, ovary, or urinary bladder cancers. Nevertheless, none of the examined SNPs were found to be associated with depression in colorectal cancer patients, including the deletion SNP of 5-HTTLPR, rs6295 in serotonin 1a receptor (HTR1a), rs6265 in BDNF, rs1360780 in FKBP5 and rs4680 in COMT. Notably, the finding on the lack of association between rs6265 and depression was in contrast to that in studies performed in breast cancer patients, where this SNP is associated with depression risk among these patients.

#### PTSD and posttraumatic stress

Four studies examined the association of SNPs in genes with the occurrence of PTSD and level of posttraumatic stress among cancer patients.^51^^,^^52^^,^^54^^,^^63^ Luo et al.^54^ reported that two SNPs (rs35753505 and rs3924999) in the neuregulin 1 (NRG1) gene were associated with increased PTSD risk, as liver cancer patients having two copies of the rare allele in their genotype were reported to be at least twice as likely to have PTSD compared to those having a wild type genotype. In another study with liver cancer patients,^52^ the rs6265 SNP of BDNF was also shown to be positively associated with PTSD risk, where carriers of the rare allele of the SNP were about three times more likely to have PTSD. However, this study also showed that another SNP in BDNF (the 11757 ​G ​> ​C SNP) was not associated with PTSD risk. Surprisingly, contradictory findings were reported for the association of the deletion SNP of 5-HTTLPR with PTSD risk among patients of different cancer types. While Chen et al.^51^ reported that papillary thyroid carcinoma patients being homozygous for the rare allele of the deletion SNP were 1.8 times more likely to have PTSD, Zerbinati et al.^63^ showed that breast cancer patients being homozygous for the wild type allele were under higher posttraumatic stress as a result of the cancer-related problems experienced by them. Nevertheless, Chen et al.^51^ also reported that the deletion SNP of 5-HTTLPR had no effect on the severity of posttraumatic stress symptoms among the papillary thyroid carcinoma patients.

A summary of the identified SNPs and the effect of their rare alleles on the risk or severity of the psychological symptoms among various types of cancer patients is presented in Table 2.

**Table 2:** A summary of the gene polymorphisms associated with anxiety, depression, and posttraumatic stress among cancer patients and the effects of their rare allele on these psychological symptoms.

Cancer types	Anxiety	Depression	PTSD/Posttraumatic stress
Breast cancer	***Genes in inflammatory pathways* rs1799964 (TNF-α)** – ↓ severity **rs3093662 (TNF-α)** – ↑ severity ***Genes in serotonergic pathway*** **Deletion SNP (5-HTTLPR)** – ↑ anxious preoccupation (smaller decrease in severity over time) ***Genes in dopamine breakdown*** **rs4680 (COMT)** – ↑ severity	***Genes in inflammatory pathways*** **−511 C > T SNP (IL-1β)** – ↑ risk **rs1800795 (IL-6)** – ↑ severity **rs2069840 (IL-6)** – ↑ severity **rs1799964 (TNF-α)** – ↓ severity **rs9376268 (IFNGR1)** – ↑ severity ***Genes in serotonergic pathway*** **Deletion SNP (5-HTTLPR)** – inconsistencies in findings between ↑ severity and no effect ***Neurotrophic genes*** **rs6265 (BDNF)** – ↑ severity and risk	***Genes in serotonergic pathway*** **Deletion SNP (5-HTTLPR)** – ↓ risk
Gastric cancer	***Neurotrophic genes*** **rs6265 (BDNF)** – ↑ severity (anxious preoccupation) ***Genes in regulation of stress response* rs1360780 (FKBP5)** – ↑ severity over time **rs9296158 (FKBP5)** – ↑ severity over time **rs9470080 (FKBP5)** – ↑ severity over time	***Genes in regulation of stress response* rs9296158 (FKBP5)** – ↑ severity over time **rs9470080 (FKBP5)** – ↑ severity over time ***Genes involved in cellular apoptosis*rs10781582 (BNIP3)** – ↓ risk	
Lung cancer		***Genes in inflammatory pathways*** **−251 ​T ​> ​A SNP (IL-8)** – ↓ risk of severe depression	
Liver cancer			***Neurotrophic genes*** **rs6265 (BDNF)** – ↑ risk ***Genes in inflammatory pathways*rs35753505 (NRG1)** – ↑ risk **rs3924999 (NRG1)** – ↑ risk
Miscellaneous	***Antioxidant genes*** **Deletion of GSTM1** – ↑ severity (medulloblastoma patients)	***Antioxidant genes*** **Deletion of GSTM1** – ↑ severity (medulloblastoma patients) ***Genes in serotonergic pathway*** **Deletion SNP (5-HTTLPR)** – ↑ risk (papillary thyroid carcinoma patients) ***Genes in regulation of stress response*** **BClI SNP (NR3C1)** – ↑ risk (acute lymphoblastic leukaemia patients)	***Genes in serotonergic pathway*** **Deletion SNP (5-HTTLPR)** – ↑ risk (Papillary thyroid carcinoma patients)

Abbreviations: 5-HTTLPR: serotonin-transporter-linked promoter region; BDNF: brain-derived neurotrophic factor; BNIP3: Bcl-2/adenovirus E1B 19 ​kDa-interacting protein 3; COMT: catechol-O-methyl transferase; FKBP5: FK506 binding protein 5; GSTM1: glutathione S-transferase Mu 1; IFNGR1: interferon-γ receptor 1; IL-1β: interleukin-1 beta; IL-6: interleukin-6; IL-8: interleukin-8; NR3C1: nuclear receptor subfamily 3 group C member 1; NRG1: neuregulin 1; PTSD: posttraumatic stress disorder; SNP: single nucleotide polymorphism; TNF-α: tumour necrosis factor-alpha.

## Discussion

In this review, we attempt to provide further information on the role of genetic polymorphisms in the occurrence of psychological issues in cancer patients, and build on that disseminated in the two previous reviews that specifically focussed on breast cancer patients.^35^^,^^36^ The most common SNPs associated with depression are those located in genes involved in pro-inflammatory pathways, where having a rare allele in the SNP of pro-inflammatory cytokines was found to contribute to modifications in depression risk and/or severity. This suggests the contributory role of these cytokines and their associated inflammatory pathways in the development of depression, and this is consistent with previous findings reviewed by Miller and Raison.^68^ Nevertheless, the association between the deletion SNP of 5-HTTLPR and depression among cancer patients appears inconsistent, potentially owing to the use of different instruments between studies for outcome assessments. Further, we had explored further potential genes that may be related to the development of anxiety, depression, and PTSD among cancer patients, and had revealed additional genes whose SNPs may also affect the development of these psychological symptoms. These genes were either involved in the regulation of stress response (FKBP5 and NR3C1), antioxidation (GSTM1), dopamine catabolism (COMT), signalling pathways contributing to inflammation (NRG1) and cellular apoptosis (BNIP3). These additionally identified genes containing SNPs that may affect the development of anxiety, depression, and PTSD may help provide information on the additional molecular pathways that can be targeted to address these psychological or psychiatric symptoms, and could potentially serve as prognostic biomarkers for these symptoms among patients of various cancer types.

There is evidence that anxiety, depression, and PTSD are correlated with each other in cancer patients. For example, anxiety and depression are known to form a symptom cluster among cancer patients.^69^^,^^70^ Further, among patients who have anxiety and depression, PTSD is one of the most prevalent comorbidities experienced by them,^71^ and it was shown to correlate with the latter two symptoms in cancer patients.^72^ It is therefore tempting to speculate that these symptoms in cancer patients could be attributed to common biological pathways, where targeting such pathways may help alleviate all these symptoms. Our review findings could potentially provide support to this hypothesis. Several included studies showed that the rs6265 SNP of the neurotrophin BDNF is associated with anxiety, depression, or PTSD in cancer patients,^42^^,^^52^^,^^58^^,^^60^ although this association was not observed in all studies examining this SNP. Here, the rare Met allele in this SNP would confer an increase in the severity or risk of these symptoms experienced by these patients. With the Met allele being responsible for leading to the reduced release of BDNF, lowered BDNF levels in the brain and subsequently brain dysfunction and memory loss,^73^^,^^74^ it is possible that these psychological symptoms experienced by cancer patients could be contributed by neuronal dysfunction. Following this line of argument, targeting pathways leading to increased brain BDNF levels could potentially serve as a symptom management strategy for treating psychological symptoms among cancer patients. Nevertheless, the molecular mechanisms of how reduced brain BDNF levels could lead to these psychological symptoms remain elusive. Research on this issue is needed for providing further clues in therapeutic development in the relief of these psychological symptoms among patients.

An interesting point for discussion regarding the association of the rs6265 SNP of BDNF with anxiety, depression, and PTSD is that such association appears to be existent only for patients who are younger. The four studies reporting the association of this SNP with these psychological symptoms of interest involved subjects who were younger, with their mean age ranging between 44 and 57 years.^42^^,^^52^^,^^58^^,^^60^ However, studies observing no such association were all having samples comprising older patients, with their mean age being over 65 years.^38^^,^^43^^,^^65^ It is unclear as to the cause of this apparent age-specific association between the rs6265 SNP and the psychological symptoms. It is possible that this age-specific association could be contributed by the higher susceptibility of older cancer patients to the development of anxiety and depression, as evidenced by the reported higher prevalence of these symptoms among older adults with cancer.^75^ This higher susceptibility would likely render a more uniform distribution of depressed subjects across various genotypes for the rs6265 SNP, thereby making the association of this SNP with these symptoms less significant. This possibility is yet largely speculative, but the observed age-specific association between rs6265 SNP and psychological symptoms in cancer patients would prompt a need for subgroup analyses of such association based on age in future studies.

A finding of note is that certain SNPs could exhibit differential effects on the risk or severity of psychological symptoms in different types of cancer patients. For example, Chen et al.^51^ showed that papillary thyroid carcinoma patients having a homozygous genotype for the rare allele of the deletion SNP of 5-HTTLPR would be predisposed to a higher risk of PTSD, although this SNP had no effect on the severity of posttraumatic stress. However, Zerbinati et al.^63^ reported that breast cancer patients with the rare allele of this SNP were at reduced risk of PTSD as a result of cancer-related problems. Likewise, while the rs6265 SNP of BDNF was associated with anxiety in gastric cancer patients,^60^ such association did not appear to exist in prostate cancer patients.^65^ Such inconsistency was also observed for the −251 ​T ​> ​A SNP of interleukin-8 on depression among breast and lung cancer patients, and the deletion SNP of 5-HTTLPR on depression among various types of cancer patients. It is likely that such discrepancies in findings could be attributed to the use of different instruments in outcome assessment between studies. However, it is also possible that the same SNP in a gene could have a different effect in different types of cancer patients. Notably, Chen et al.^51^ and Zerbinati et al.^63^ both used impact of event scale for outcome measurements in different cancer patient types, yet different outcomes were observed as described above. It is therefore possible that a particular SNP could exert different effects on different types of cancer patients in terms of their psychological outcomes. Given the paucity of studies on a particular genetic variation on the psychological symptoms in various types of cancer patients, further research in this area is recommended.

We acknowledge three limitations of this review. First, although we attempted to enhance the comprehensiveness of the review by including articles published in Chinese, studies published in languages other than English and Chinese were not included. Second, there is heterogeneity in demographic and clinical characteristics of study samples between the included studies, including cancer type, age, and ethnicity. Heterogeneity in these factors would likely have an influence on the significance of the association between the genetic polymorphisms and the examined psychological symptoms, the outcome of interest in this review. In particular, only a few studies would examine the association of a particular SNP with a particular psychological symptom. Third, the majority of the studies reported such association based on self-report data on patients' psychological outcomes, and there are variations in the instruments used for such outcome assessments. These self-report data may have limited the reflection of the true effects of the SNPs on patients' psychological outcomes. These factors prompt caution in the interpretation of the findings of this review.

## Conclusions

With the growing importance of personalised care that aims to reduce comorbidities as a result of cancer treatment,^76^ a better understanding of the effect of various genetic variations on the risk and severity of cancer-associated symptoms is required for the development of personalised plans for patients bearing these genetic variations to manage these symptoms effectively. Our review findings could provide further information on this issue, by summarising our current knowledge on the SNPs that affect the development of anxiety, depression, and PTSD, the three common psychological symptoms experienced by cancer patients. Overall, there is evidence showing that SNPs present in genes involved in various metabolic and cellular processes including inflammation, serotonergic signalling, regulation of stress response, antioxidation, dopamine catabolism, and apoptosis could potentially modify the risk and/or severity of these psychological symptoms in cancer patients. Patients found to bear these SNPs through genetic testing may be given more attention to their psychological health, through the provision of more intensive psychological care and the enhancement of its access. Nevertheless, studies on SNPs in certain genes on anxiety, depression, and PTSD are still scarce. Most of them were conducted among breast cancer patients, resulting in scarce data on the association of genetic SNPs with these symptoms in patients of other cancer types. Moreover, gene polymorphisms examined by multiple studies yielded inconsistent results, due to either the use of different instruments between studies for outcome assessments or even the potential variations in the effect of SNPs on patients of different cancer types. Further research on this matter, with the use of standardised instruments for outcome assessments and stratification of subjects based on their age, is recommended, which provide stronger evidence for the association of SNPs in various genes and psychological symptoms in cancer patients.

## Declaration of competing interest

One of the authors of the manuscript, Winnie K.W. So, is the editor-in-chief of the Asia-Pacific Journal of Oncology Nursing. The article was subject to the journal's standard procedures, with peer review handled independently of Prof. So and their research groups.

## Funding

Nil.
