# Supplementary material for: Association of genetic polymorphisms with psychological symptoms in cancer: A systematic review
Source: Asia Pac J Oncol Nurs. 2021 Dec 25;9(1):12–20. doi: 10.1016/j.apjon.2021.11.006 (PMC9072184; doi:10.1016/j.apjon.2021.11.006)
Supplement: Multimedia component 1 [file mmc1.docx]

**Supplementary Tables**

**Table S1. The search strategy used for literature search in PubMed, PsycINFO, CINAHL, OVID MEDLINE, Web of Science**

| “cancer” OR “neoplasm*” OR “carcinoma” OR “malignan*” OR “tumor*” OR “tumour*” |
| --- |
| **AND** |
| “genetic variant*” OR “genomic variant*” OR “allelic variant*” OR “genetic variation*” OR “genomic variation*” OR “polymorphism*” OR “SNP” OR “SNPs” OR “GWAS” OR “genome-wide association study” OR “genome-wide association studies” |
| **AND** |
| “psychological” OR “psychiatric” OR “symptom*” OR “anxiety” OR “anxious” OR “depression” OR “depressive” OR “stress disorder” OR “posttraumatic stress” OR “post-traumatic stress” |

**Table S2. The search strategy used for literature search in Chinese databases (Wanfang, CNKI, CQVIP and SinoMed**

| “癌” OR “肿瘤”` |
| --- |
| **AND** |
| “基因” OR “基因多态性” |
| **AND** |
| “心理” OR “焦虑” OR”抑郁” OR “创伤后压力” |

**Table S3. Characteristics of the included studies and their major relevant findings**

| **Author / year / Location of study** | **Sample characteristics / sample size / source of sample** | **Gene polymorphisms examined for association with anxiety, depression or PTSD / name of gene where the gene polymorphisms are located** | **Psychological symptoms explored** | **Instruments / methodologies for symptom assessment** | **Major findings** |
| --- | --- | --- | --- | --- | --- |
| Bower et al., 2013 (40); United States | Breast cancer patients having completed curative treatment;  N = 171  Mean age: 51.5 years  Age range: 31-66 years  Eligible participants identified through a local registry | ***Interleukin-6***  IL6 -174 G>C polymorphism (rs1800795)  (Genotypes: GG, GC, CC) | - Depression | Beck Depression  Inventory-II | **IL6-174 G>C polymorphism is associated with depression among patients**  Patients bearing the GG genotype experienced higher severity of depression compared to those bearing the CC and GC genotype (*p* = 0.024)  ***Mean depression score for each genotype***  GG genotype: 9.74  CC genotype: 7.66  GC genotype: 7.83 |
| Brackett et al., 2012 (41); United States | Paediatric brain cancer (medulloblastoma) patients having completed treatment for at least 5 years;  N = 109  Age at diagnosis: 8.0 ± 0.41 years  Subjects were participants of a previous Childhood Cancer Survivor Study conducted locally | ***Glutathione S-transferase Mu 1 (GSTM1)***  Polymorphism of GSTM1 leading to gene deletion  (rs number not reported)  (Genotype: null, non-null) | - Anxiety - Depression | Brief Symptom Inventory–18 (BSI-18) | **GSTM1 polymorphism leading to its gene deletion (GSTM1-null) is associated with anxiety and depression among the paediatric medulloblastoma survivors**  ***Anxiety***  Patients having the GSTM1 null genotype had higher mean score for anxiety compared to those having the GSTM1 non-null genotype (*p* = 0.04)  GSTM1 null: 47.3 ± 9.17  GSTM1 non-null: 43.9 ± 7.76  ***Depression***  Patients having the GSTM1 null genotype had higher mean score for depression compared to those having the GSTM1 non-null genotype (*p* = 0.03)  GSTM1 null: 51.0 ± 9.83  GSTM1 non-null: 47.0 ± 9.36  **Multivariate regression analysis demonstrated that the GSTM1 null genotype remain a significant predictor of anxiety and depression scores among patients, after controlling for age, sex, and radiation dose**  ***Anxiety***  GSTM1 null: Reference  GSTM1 non-null: Estimate = -3.29; *p* = 0.05  ***Depression***  GSTM1 null: Reference  GSTM1 non-null: Estimate = -3.85; *p* = 0.04 |
| Chen et al., 2019 (51); China | Patients with papillary thyroid carcinoma who had completed surgery;    N = 287  Age: 45.15 ± 10.25 years  Subjects were recruited in a local hospital | ***Serotonin-transporter-linked polymorphic region (5-HTTLPR) gene***  Deletion polymorphism in the polymorphic region of serotonin transporter gene leading to a shorter version of the gene  (rs number not reported)  (Genotypes: SS, SL, LL) | - Posttraumatic stress symptom severity and PTSD risk - Depression | ***PTSD risk***  The diagnostic criteria of the Diagnostic and Statistical Manual of Mental Disorders-IV  ***Proxy measure for posttraumatic stress symptom severity*** Impact of Event Scale-Revised Edition (IES-R)  ***Depression***  Hamilton Depression Scale (HAMD) | **5-HTTLPR polymorphism is associated with PTSD risk**  Distribution frequency of patients having the homozygous short (SS) genotype in the PTSD group is higher, while that of patients having are either heterozygous (SL) or homozygous long (LL) genotypes in the non-PTSD group is higher. (OR: 1.829; 95% CI: 1.104 – 3.030; *p* = 0.018)  ***Patients with SS genotype***  PTSD group: 40.2%  Non-PTSD group: 26.9%  ***Patients with SL or LL genotype***  PTSD group: 59.8%  Non-PTSD group: 73.1%  (SS and SL genotypes would lead to reduced expression level of serotonin transporter)  **5-HTTLPR polymorphism is not associated with higher posttraumatic stress symptom severity among cancer patients with PTSD**  There was no significant difference between papillary thyroid carcinoma patients with PTSD with SS genotype and those with SL+LL genotype in their distribution in various levels of posttraumatic stress symptom severity based on the IES-R score. This phenomenon was observed for both patients with PTSD (*p* = 0.849) and patients without PTSD (*p* = 0.67)  ***Patients with PTSD***  ***Patients with SS genotype***  Mild symptom: 35.6%  Moderate symptom: 31.1%  Severe symptom: 33.3%  ***Patients with SL or LL genotype***  Mild symptom: 37.3%  Moderate symptom: 34.3%  Severe symptom: 28.4%  ***Patients without PTSD***  ***Patients with SS genotype***  Mild symptom: 93.6%  Moderate symptom: 6.4%  Severe symptom: 0.0%  ***Patients with SL or LL genotype***  Mild symptom: 97.7%  Moderate symptom: 2.3%  Severe symptom: 0.0%  **5-HTTLPR polymorphism is associated with higher depression among cancer patients with PTSD and those without PTSD**  Significantly more patients with SS genotype are distributed in a higher HAMD score group (having a HAMD score of >20) compared to those with SL or LL genotype, both among patients with PTSD (*p* = 0.04) and those without PTSD (*p* < 0.001)  ***Patients with PTSD***  ***Patients with SS genotype***  HAMD score < 20: 6.7%  HAMD score > 20: 93.3%  ***Patients with SL or LL genotype***  HAMD score < 20: 20.9%  HAMD score > 20: 79.1%  ***Patients without PTSD***  ***Patients with SS genotype***  HAMD score < 20: 78.7%  HAMD score > 20: 21.3%  ***Patients with SL or LL genotype***  HAMD score < 20: 96.1%  HAMD score > 20: 3.9% |
| Cihan et al., 2017 (66); Turkey | Paediatric patients with acute lymphoblastic leukemia who were undergoing treatment;    N = 47 (induction phase – treatment with prednisolone)  N = 44 (reinduction phase – treatment with dexamethasone)  Mean age: 7.0 years  Subjects were recruited in a local hospital | ***Nuclear Receptor Subfamily 3 Group C Member 1 (NR3C1)***  The Bcl*I* polymorphism (C>G polymorphism at intron 2 of the NR3C1 gene)  (rs number not reported)  (Genotypes: CC, CG, GG) | - Depression symptoms | National Cancer Institute’s Common Terminology Criteria  for Adverse Events, version 4.0 | **The presence of G allele in NR3C1 (The Bcl*I* polymorphism) is associated with higher frequency of depression among patients**  Depression is more frequent among patients having the G allele in NR3C1 (i.e. bearing the CG or GG genotype), compared to those having the homozygous CC genotype during both the induction phase (OR = 5.5; *p* = 0.031) and reinduction phase (OR = 5.2; *p* = 0.04).  ***Induction phase (treatment with prednisolone)***  ***Patients with CC genotype***  Depressed: 10.5%  No depression: 89.5%  ***Patients with CG+GG genotype (carriers of G allele)***  Depressed: 39.3%  No depression: 60.7%  ***Reinduction phase (treatment with dexamethasone)***  ***Patients with CC genotype***  Depressed: 11.8%  No depression: 88.2%  ***Patients with CG+GG genotype (carriers of G allele)***  Depressed: 40.7%  No depression: 59.3% |
| Dai et al., 2008 (55); China | Breast cancer patients, 2-4 weeks post-surgery prior to chemotherapy  N = 115  Age not reported  Subjects recruited at a breast care centre of a university affiliated hospital | ***Serotonin-transporter-linked polymorphic region (5-HTTLPR) gene***  Deletion polymorphism in the polymorphic region of serotonin transporter gene leading to a shorter version of the gene  (rs number not reported)  (Genotypes: SS, SL, LL) | - Depression | Hamilton Depression Rating Scale (HAMD) | **The 5-HTTLPR gene polymorphisms were associated with the severity of depression**  Patients bearing three alleles had significantly different depression scores. Those with the LL genotypes had significantly lower depression score compared to those having the SS genotype.  ***Mean HAMD score (standard deviation) for patients with various 5-HTTLPR genotypes***  LL – 6.33 ± 6.22  SL – 9.07 ± 8.03  SS – 17.1 ± 9.88  (*p* = 0.000)  ***Comparison of mean HAMD score (standard deviation) for various 5-HTTLPR genotypes based on serotonin transporter expression level***  Normal expression level (genotype: LL) – 6.33 ± 6.22  Lower expression level (genotype: SL and SS) – 13.72 ± 9.93  (*p* = 0.004) |
| Dooley et al., 2016 (42); United States | Breast cancer patients having completed treatment within the past 3 months  N = 112  Age: 51.4 ± 8.9 years (Val/Val genotype)  and 51.2 ± 7.0 years (for Val/Met and Met/Met genotypes)  Eligible participants identified through a local registry (Subjects were participants of a previous study) | ***Brain-derived neurotrophic factor (BDNF)***  The Val66Met polymorphism – BDNF 196 G>A  (rs6265)  (Genotypes: GG, GA, AA) | - Depression | Beck Depression Inventory-II (BDI-II) | **Val66Met polymorphism in BDNF is associated with depression severity (as measured by BDI-II cognitive subscale score) via the increased production of C-reactive protein (CRP)**   - Significant interaction was found between CRP level and BDNF genotype, and this interaction was demonstrated to be related to the severity of depression. (β coefficient = 0.72, t value = 2.94, *p* = 0.004) - Among patients with Val/Val homozygous genotype, there was no significant relationship between CRP level and severity of depression. Such relationship tended to be negative (β coefficient = -0.21, t value = -1.87, *p* = 0.07) - Among patients with Val/Met or Met/Met genotype (i.e. Met carriers), there was a positive and significant association between CRP and severity of depression (β coefficient = 0.52, t value = 2.23, *p* = 0.028) |
| Eberhard et al., 2010 (39); Sweden | Testicular germ cell cancer having completed treatment  N = 140  Age of the overall sample of subjects was not reported.  Subjects were recruited in a local university hospital | ***Androgen receptor gene***  Polymorphisms leading to the length of CAG repeats and GGN repeats  (Number of CAG repeats: <20, 20-21, 22-23 and >23; Number of GGN repeats: <23, 23 and >23) | - Anxiety | Hospital Anxiety–Depression Scale (HADS) | **Number of CAG and GGN repeats in the androgen receptor is not associated with anxiety**  The number of CAG repeats had no effect on the anxiety risk among the subjects (OR: 1.1; 95% CI: 0.94 - 1.30; *p* = 0.24)  The number of GGN repeats had no effect on anxiety risk of the patients either (OR and 95% CI not reported; *p* = 0.23) |
| Feng et al., 2020 (43); United States | Patients with any type of cancer undergoing any type of cancer treatment  N = 180  (91% prostate cancer patients)  Age: 65.3 ± 8.1 years  Retrospective analysis of patient data from previous studies | ***Brain-derived neurotrophic factor (BDNF)***  The Val66Met polymorphism –BDNF 196 G>A  (rs6265)  (Genotypes: GG, GA, AA) | - Depression | Hamilton Depression Scale (HAMD) | **Val66Met polymorphism in BDNF is not associated with depression risk and severity**   - There was no significant difference between the proportion of depressed patients with Val/Val genotype and that of depressed patients who are Met carriers (1.64% vs 2.54%; *p* = 0.882) - Mean HAMD score of patients with Val/Val homozygous genotype is not significantly different from that of patients who are Met carriers (i.e. Val/Met or Met/Met genotype) (1.508 ± 2.087 vs 1.492 ± 2.171; *p* = 0.961) - Regression analysis also demonstrated that Carrying a Met allele does not predict depression risk (Estimate: 0.0063; 95% CI: -0.2726 – 0.2852; *p* = 0.97) |
| Gilbert et al., 2012 (44); United States | Head and neck cancer patients having completed cancer treatment  N = 33  Age: 55.2 ± 9.8 years  Subjects recruited at a cancer clinic of a local university medical centre | ***Serotonin-transporter-linked polymorphic region (5-HTTLPR) gene***  Deletion polymorphism in the polymorphic region of serotonin transporter gene leading to a shorter version of the gene  (rs number not reported)  (Genotypes: SS, SL, LL) | - Depression | Diagnosis through the Structured Clinical Interview for Diagnostic and Statistical Manual of Mental Disorders | **Having a short allele of the 5-HTTLPR gene (i.e. Short allele carriers) were more likely to have depression compared to patients bearing LL genotype, but the increase in likelihood was not significant**   - 85.7% patients having the short allele had depression, as opposed to 68.4% of these short allele carriers who did not have depression (*p* = 0.252) - Carrying at least one short allele of the 5-HTTLPR gene (leading to reduced expression of serotonin transporter) would confer an increased likelihood of having depression, but this increase was not significant (OR: 2.77; *p* = 0.263) |
| Grassi et al., 2010 (61); Italy | Breast cancer patients undergoing treatment;  N = 145  Age: 55.9 ± 9.0 years  Subjects recruited at clinics and day-hospital services of a local university hospital | ***Serotonin-transporter-linked polymorphic region (5-HTTLPR) gene***  Deletion polymorphism in the polymorphic region of serotonin transporter gene leading to a shorter version of the gene  (rs number not reported)  (Genotypes: SS, SL, LL) | - Depression | Hospital Anxiety–Depression Scale (HADS) | **Polymorphism in 5-HTTLPR gene is not associated with depression**   - No significant difference in HADS score between the various genotypes (SS, SL and LL) - No significant association was observed between the genotypes of 5-HTTLPR and level of depression severity among patients (β coefficient = 1.07; Standard error = 0.44; *p* = 0.87) - There was also no significant association between genotypes of 5-HTTLPR and proportion of depressed cases among patients (β coefficient = 0.8; Standard error = 0.44; *p* = 0.87) |
| Guo et al., 2019 (52); China | Hospitalised patients with liver cancer  N = 248  Age: 44.0 ± 7.7 years (patients with PTSD) and 44.5 ± 7.9 years (patients without PTSD)  Subjects recruited at a local hospital | ***Brain-derived neurotrophic factor (BDNF)***  The Val66Met polymorphism –BDNF 196 G>A  (rs6265)  (Genotypes: GG, GA, AA)  BDNF 11757 G>C polymorphism (rs number not reported)  (Genotypes: GG, GC, CC) | - Posttraumatic stress | Diagnosis of PTSD through the Diagnostic and Statistical Manual  fifth edition | **The presence of the A allele in the rs6265 polymorphism led to higher PTSD risk**  Compared to patients having the GG genotype, patients bearing genotypes having the presence of the A allele at position 196 of BDNF (rs6265), i.e. GA or AA, were at about 3 times higher risk of PTSD.   - GG – Reference - GA – OR: 2.790; 95% CI: 1.400 – 5.560; *p* = 0.003 - AA – OR: 3.477; 95% CI: 1.576 – 7.671; *p* = 0.002 - GA+AA – OR: 2.984; 95% CI: 1.539 – 5.788; *p* < 0.001   **However, the BDNF 11757 G>C polymorphism is not associated with PTSD risk**  There was no significant difference in the odds of having PTSD among patients bearing the GG, GC or CC genotypes   - GG – Reference - GC – OR: 0.935; 95% CI: 0.510 – 1.716; *p* = 0.829 - CC – OR: 0.729; 95% CI: 0.339 – 1.568; *p* = 0.418 - GC+CC – OR: 0.876; 95% CI: 0.490 – 1.567; *p* = 0.655 |
| Kang et al., 2012 (56); South Korea | Gastric cancer patients undergoing chemotherapy  N = 130 (at baseline)  N = 93 (at follow-up)  Age: 58.1 ± 11.6 years  Subjects recruited at clinics in a local cancer centre and a local hospital | ***FK506 binding protein 5 (FKBP5)***  A C>T polymorphism (rs1360780)  (Genotypes: CC, CT, TT)  A G>A polymorphism (rs9296158)  (Genotypes: GG, GA, AA)  Another C>T polymorphism (rs9470080)  (Genotypes: CC, CT, TT) | - Depression - Anxiety | Hospital Anxiety and Depression Scale (HADS) | **Some FKBP5 polymorphisms are associated with anxiety and depression among cancer patients under prolonged stress**  One-way repeated measures analysis of variance (ANOVA) was used to determine whether there are significant difference in the change of anxiety and depression score over time (between at diagnosis and six weeks after diagnosis) between the homozygous and heterozygous genotypes in all three gene polymorphisms.  ***rs1360780***  ***Anxiety***   - No significant difference in anxiety score between the CC, CT and TT genotypes (F = 2.00, *p* = 0.14) - The gene polymorphism had no significant time effect on anxiety score (F = 0.45, *p* = 0.50) - A genotype by time interaction occurred for this gene polymorphism on anxiety score (F = 3.39, *p* = 0.038)   ***Depression***   - A significant difference was observed for depression score between the CC, CT and TT genotypes (F = 3.71, *p* = 0.028) - There was a trend for a significant effect of time on depression score for this gene polymorphism (F = 3.25, *p* = 0.075) - No genotype by time interaction for this gene polymorphism on depression score (F = 1.93, *p* = 0.15)   ***rs9296158***  ***Anxiety***   - No significant difference in anxiety score between the GG, GA and AA genotypes (F = 0.65, *p* = 0.52) - The gene polymorphism had no significant time effect on anxiety score (F = 2.34, *p* = 0.13) - However, this gene polymorphism had a significant genotype by time interaction effect on anxiety score (F = 4.38, *p* = 0.015)   ***Depression***   - No significant difference in depression score between the GG, GA and AA genotypes (F = 2.27, *p* = 0.11) - There was a strong and significant effect of time on depression score for this gene polymorphism (F = 7.85, *p* = 0.006) - Genotype by time interaction effect was observed for this gene polymorphism on depression score (F = 3.57, *p* = 0.032)   ***rs9470080***  ***Anxiety***   - No significant difference in anxiety score between the CC, CT and TT genotypes (F = 1.38, *p* = 0.26) - The gene polymorphism had no significant time effect on anxiety score (F = 2.14, *p* = 0.15) - However, this gene polymorphism had a significant genotype by time interaction effect on anxiety score (F = 3.95, *p* = 0.023)   ***Depression***   - No significant difference in depression score between the GG, GA and AA genotypes (F = 1.78, *p* = 0.17) - There was a strong and significant effect of time on depression score for this gene polymorphism (F = 7.85, *p* = 0.007) - Genotype by time interaction effect was observed for this gene polymorphism on depression score (F = 3.79, *p* = 0.026)   **rs9470080 and rs9296158 were significant predictors of anxiety and depression respectively at six weeks after diagnosis**   - rs9470080 on anxiety score – Coefficient of determination = 0.26; F = 16.1; *p* < 0.001 - rs9296158 on depression score – Coefficient of determination = 0.28; F = 11.6; *p* < 0.001 |
| Kim et al., 2012 (57); South Korea | Breast cancer patients; stage of treatment is not reported  N = 186  Age: 54.1 ± 8.7 years (depressed patients) and 54.0 ± 8.5 years (non-depressed patients)  Subjects recruited at a cancer centre of a local hospital | ***Serotonin-transporter-linked polymorphic region (5-HTTLPR) gene***  Deletion polymorphism in the polymorphic region of serotonin transporter gene leading to a shorter version of the gene  (rs number not reported)  (Genotypes: SS, SL, LL) | - Depression | Hospital Anxiety and Depression Scale (HADS)  Hamilton Depression Scale (HAMD) | **5-HTTLPR gene polymorphism is not associated with occurrence of depression**  There was no significant difference in the distribution of the patients bearing the various genotypes (SS, SL and LL) in the depressed and non-depressed groups. (chi-squared value: 1.094; *p* = 0.579)  ***Proportion of depressed patients bearing the following genotypes:***  SS: 53.1%  LS: 44.9%  LL: 2.0%  ***Proportion of non-depressed patients bearing the following genotypes:***  SS: 55.5%  LS: 39.4%  LL: 5.1%  **However, 5-HTTLPR gene polymorphism is associated with severity of depression among patients with poor body image and sexual function**   - Patients having the S allele (having genotype of SS or SL) had higher depression severity compared to those with the LL genotype (F = 7.59, *p* = 0.047) - Patients with poor body image/sexual function and bearing LL genotype experienced lower depression severity, while those with poor body image/sexual function but bearing the SS or SL genotypes experienced higher depression severity. - 5-HTTLPR gene polymorphism predicts depression severity via the interaction effect between genotype and body image/sexual function of patients.   (SS and SL genotypes lead to reduced expression level of serotonin transporter) |
| Kim et al., 2012 (58); South Korea | Breast cancer patients who were undergoing mastectomy  N = 309 (at baseline)  N = 244 (at follow-up)  Age: 50.8 ± 9.7 years  Subjects recruited at a clinic of a local university hospital (Subjects were participants of a previous study) | ***Serotonin-transporter-linked polymorphic region (5-HTTLPR) gene***  Deletion polymorphism in the polymorphic region of serotonin transporter gene leading to a shorter version of the gene  (rs number not reported)  (Genotypes: SS, SL, LL)  ***Serotonin 2a receptor (5-HTR2a) gene***  5HTR2a 1438 A>G polymorphism  (rs number not reported) (Genotypes: AA, AG, GG)  5HTR2a 102 T>C polymorphism (rs number not reported)  (Genotypes: TT, TC, CC)  ***Serotonin transporter intron 2 – variable number tandem repeat (STin2 VNTR)***  Genetic variant of having 10 repeats or 12 repeats  (Genotypes: 10/10, 10/12, 12/12)  ***Brain-derived neurotrophic factor (BDNF)***  The Val66Met polymorphism –BDNF 196 G>A  (rs6265)  (Genotypes: Val/Val, Val/Met, Met/Met) | - Depression | Diagnosis through Diagnostic and Statistical Manual of Mental Disorders – Fourth edition (DSM-IV) | **None of the gene polymorphisms related to the 5-HTR2a, nor variants in 5-HTTLPR and STin2, are associated with depression risk**  ***5-HTTLPR deletion polymorphism***  ***Prevalent depression (p = 0.55)***  LL – Reference  SL – AOR: 1.58; 95% CI: 0.50-5.05  SS – AOR: 1.18; 95% CI: 0.38-3.67  ***Persistent depression (p = 0.22)***  LL – Reference  SL – AOR: 1.07; 95% CI: 0.11-10.10  SS – AOR: 0.35; 95% CI: 0.03-3.71  ***Incident depression (p = 0.26)***  LL – Reference  SL – AOR: 3.46; 95% CI: 0.42-28.80  SS – AOR: 1.86; 95% CI: 0.23-15.30  (SS and SL genotypes lead to reduced expression level of serotonin transporter)  ***5HTR2a 1438 A>G polymorphism***  ***Prevalent depression (p = 0.69)***  GG – Reference  GA – AOR: 0.94; 95% CI: 0.45-1.98  AA – AOR: 0.75; 95% CI: 0.38-1.48  ***Persistent depression (p = 0.62)***  GG – Reference  GA – AOR: 2.10; 95% CI: 0.44-10.10  AA – AOR: 1.32; 95% CI: 0.26-6.65  ***Incident depression (p = 0.96)***  GG – Reference  GA – AOR: 0.86; 95% CI: 0.28-2.67  AA – AOR: 0.89; 95% CI: 0.34-2.38  ***5HTR2a 102 T>C polymorphism***  ***Prevalent depression (p = 0.77)***  TT – Reference  TC – AOR: 1.23; 95% CI: 0.60-2.53  CC – AOR: 1.31; 95% CI: 0.61-2.84  ***Persistent depression (p = 0.58)***  TT – Reference  TC – AOR: 1.67; 95% CI: 0.36-7.79  CC – AOR: 0.73; 95% CI: 0.12-4.36  ***Incident depression (p = 0.49)***  TT – Reference  TC – AOR: 1.95; 95% CI: 0.60-6.35  CC – AOR: 2.05; 95% CI: 0.57-7.28  ***STin2 VNTR***  ***Prevalent depression (p = 0.57)***  10/10 or 10/12 – Reference  12/12 – AOR: 0.80; 95% CI: 0.37-1.73  ***Persistent depression (p = 0.91)***  10/10 or 10/12 – Reference  12/12 – AOR: 0.91; 95% CI: 0.19-4.43  ***Incident depression (p = 0.91)***  10/10 or 10/12 – Reference  12/12 – AOR: 1.08; 95% CI: 0.30-3.91  **The Val66Met polymorphism is associated with risk of prevalent and persistent depression, but not that of incident depression.**  Patients bearing the homozygous Met/Met genotype are at increased risk of prevalent and persistent depression.  ***The Val66Met polymorphism***  ***Prevalent depression (p = 0.018)***  Val/Val – Reference  Val/Met – AOR: 1.03; 95% CI: 0.48-2.19  Met/Met – AOR: 2.63; 95% CI: 1.12-6.14  ***Persistent depression (p = 0.003)***  Val/Val – Reference  Val/Met – AOR: 0.54; 95% CI: 0.08-3.56  Met/Met – AOR: 8.07; 95% CI: 1.26-51.60  ***Incident depression (p = 0.19)***  Val/Val – Reference  Val/Met – AOR: 0.59; 95% CI: 0.18-1.87  Met/Met – AOR: 0.38; 95% CI: 0.13-1.09 |
| Kim et al., 2013 (59); South Korea | Breast cancer patients who were undergoing mastectomy  N = 309 (at baseline)  N = 244 (at follow-up)  Age: 50.8 ± 9.7 years  Subjects recruited at a clinic of a local university hospital (Subjects were participants of a previous study) | ***Tumour necrosis factor***  TNF-α -850 C>T polymorphism  (rs number not reported)  (Genotype: CC, CT, TT)  TNF-α -308 G>A polymorphism  (rs number not reported)  (Genotype: GG, GA, AA)  ***Interleukin-1β***  IL-1β -511 C>T polymorphism (rs number not reported)  (Genotypes: CC, CT, TT)  IL-1β +3953 C>T polymorphism (rs number not reported)  (Genotypes: CC, CT, TT)  ***Interleukin-8***  IL-8 -251 T>A polymorphism  (rs number not reported)  (Genotypes: TT, TA, AA)  ***Interleukin-4***  IL-4 +33 T>C polymorphism  (rs number not reported)  (Genotypes: TT, TC, CC)  ***Interleukin-10***  IL-10 -1082 G>A polymorphism  (rs number not reported)  (Genotypes: GG, GA, AA) | - Depression | Diagnosis through Diagnostic and Statistical Manual of Mental Disorders – Fourth edition (DSM-IV) | **The IL-1β -511 C>T polymorphism is associated with the risk of prevalent depression and persistent depression, but not that of incident depression. All other examined polymorphisms are not associated with depression risk.**  Patients bearing the TT genotype of this polymorphism are at higher risk of prevalent depression and persistent depression.  ***TNF-α -850 C>T polymorphism***  ***Prevalent depression***  CC – Reference  CT or TT – AOR: 1.62; 95% CI: 0.91-2.88  ***Persistent depression***  CC – Reference  CT or TT – AOR: 1.52; 95% CI: 0.47-4.89  ***Incident depression***  CC – Reference  CT or TT – AOR: 0.35; 95% CI: 0.11-1.06  ***TNF-α -308 G>A polymorphism***  ***Prevalent depression***  GG – Reference  GA or AA – AOR: 1.34; 95% CI: 0.68-2.62  ***Persistent depression***  GG – Reference  GA or AA – AOR: 0.78; 95% CI: 0.16-3.93  ***Incident depression***  GG – Reference  GA or AA – AOR: 0.45; 95% CI: 0.13-1.59  ***IL-1β -511 C>T polymorphism***  ***Prevalent depression***  CC – Reference  CT – AOR: 1.52; 95% CI: 0.69-3.37  **TT – AOR: 2.34; 95% CI: 1.02-5.43 (*p* < 0.05)**  ***Persistent depression***  CC – Reference  CT – AOR: 2.38; 95% CI: 0.30-18.80  **TT – AOR: 9.95; 95% CI: 1.21-81.50 (*p* < 0.05)**  ***Incident depression***  CC – Reference  CT – AOR: 1.36; 95% CI: 0.46-4.07  TT – AOR: 1.71; 95% CI: 0.50-5.85  ***IL-1β +3953 C>T polymorphism***  ***Prevalent depression***  CC – Reference  CT – AOR: 1.71; 95% CI: 0.52-5.59  ***Persistent depression***  CC – Reference  CT – No incident depression among patients with this genotype  ***Incident depression***  CC – Reference  CT – AOR: 0.72; 95% CI: 0.09-5.92  ***IL-8 -251 T>A polymorphism***  ***Prevalent depression***  TT – Reference  TA – AOR: 1.12; 95% CI: 0.61-2.06  AA – AOR: 2.04; 95% CI: 0.79-5.23  ***Persistent depression***  TT – Reference  TA – AOR: 1.36; 95% CI: 0.35-5.26  AA – AOR: 3.96; 95% CI: 0.59-26.80  ***Incident depression***  TT – Reference  TA – AOR: 1.05; 95% CI: 0.45-2.46  AA – No incident depression among patients with this genotype  ***IL-4 +33 T>C polymorphism***  ***Prevalent depression***  TT – Reference  TC – AOR: 0.96; 95% CI: 0.52-1.77  CC – AOR: 1.48; 95% CI: 0.42-5.23  ***Persistent depression***  TT – Reference  TC – AOR: 1.78; 95% CI: 0.51-6.17  CC – AOR: 1.20; 95% CI: 0.08-17.00  ***Incident depression***  TT – Reference  TC – AOR: 0.95; 95% CI: 0.37-2.44  CC – AOR: 2.25; 95% CI: 0.42-12.10  ***IL-10 -1082 G>A polymorphism***  ***Prevalent depression***  GG – Reference  GA – AOR: 0.97; 95% CI: 0.17-5.73  AA – AOR: 1.36; 95% CI: 0.28-6.64  ***Persistent depression***  None of the patients having the GG genotype has got persistent depression  ***Incident depression***  GG – Reference  GA – AOR: 0.53; 95% CI: 0.03-9.50  AA – AOR: 1.50; 95% CI: 0.18-12.40 |
| Kim et al., 2018 (45); United States | Patients with advanced breast cancer before having curative treatment  N = 95  Age: 57.7 ± 7.4 years  Subjects were recruited at breast cancer clinics of a local university | ***Serotonin-transporter-linked polymorphic region (5-HTTLPR) gene***  Deletion polymorphism in the polymorphic region of serotonin transporter gene leading to a shorter version of the gene  (rs number not reported)  (Genotypes: SS, SL, LL) | - Depression | Centre for Epidemiologic Studies Depression scale  (CES-D) | **The presence of more S allele (i.e. SS genotype) for the 5-HTTLPR polymorphism is associated with higher depression levels**   - β-coefficient: 0.20 - *p* < 0.042 |
| Koh et al., 2014 (60); South Korea | Patients with advanced gastric cancer undergoing chemotherapy  N = 91  Age: 57.7 ± 11.4 years  Subjects recruited at a local cancer centre and an oncology clinic of a local hospital | ***Brain-derived neurotrophic factor (BDNF)***  The Val66Met polymorphism –BDNF 196 G>A  (rs6265)  (Genotypes: Val/Val, Val/Met, Met/Met) | - Anxiety | Mini-Mental Adjustment to Cancer (Mini-MAC) scale | **Met allele carriers for the Val66Met polymorphism had significantly higher level of anxiety compared to patients with Val/Val genotype.**   - Mean score for anxious preoccupation domain of the Mini-MAC scale   - Val/Val: 18.7 ± 5.7   - Val/Met and Met/Met: 21.2 ± 4.6   - *p* = 0.020 |
| Lou et al., 2021 (53); China | Gastric cancer patients. Treatment status not reported  N = 150  Mean age: 61.2 years  Subjects recruited from a local university hospital | ***Bcl-2/adenovirus E1B 19 kDa-interacting protein 3 (BNIP3)***  rs10781582  (The T>A polymorphism)  (Genotypes: TT, TA, AA)  rs3793742  (The C>T polymorphism)  (Genotypes: CC, CT, TT)  ***Death-associated protein kinase 1 (DAPK1)***  rs1329600  (The A>G polymorphism)  (Genotypes: AA, AG, GG) | - Depression - Anxiety | Hamilton Anxiety Rating Scale (HAMA)  Hamilton Depres-  sion Rating Scale (HAMD) | **The rs10781582 polymorphism of the BNIP3 gene is associated with depression, but not the rs3793742 polymorphism**  ***rs10781582***   - This polymorphism led to a decrease in the depression risk of patients   - AOR: 0.276; 95% CI: 0.128 – 0.595; *p* = 0.003   ***rs3793742***   - This polymorphism did not lead to any changes in depression risk of patients   - AOR: 1.175; 95% CI: 0.557 – 2.476; *p* = 0.672   **The rs1329600 polymorphism of DAPK1 is not associated with depression**   - AOR: 0.734; 95% CI: 0.330 – 1.632; *p* = 0.672   **All tested gene polymorphisms of BNIP3 and DAPK1 are not associated with anxiety**  ***rs10781582 (BNIP3)***   - AOR: 0.875; 95% CI: 0.433 – 1.767; *p* = 0.889   ***rs3793742 (BNIP3)***   - AOR: 0.911; 95% CI: 0.457 – 1.819; *p* = 0.889   ***rs1329600 (DAPK1)***   - AOR: 1.054; 95% CI: 0.504 – 2.204; *p* = 0.889 |
| Luo et al., 2020 (54); China | Patients with hepatocellular carcinoma (liver cancer). Treatment stage not reported  N = 234  Age: 64.0 ± 7.5 years (patients with PTSD) and 65.5 ± 8.2 years (patients without PTSD)  Subjects recruited at a local hospital | ***Neuregulin 1 (NRG1)***  rs35753505  (The T>C polymorphism)  (Genotypes: TT, TC, CC)  rs3924999  (The A>G polymorphism)  (Genotypes: AA, AG, GG) | - Posttraumatic stress | Diagnosis through Diagnostic and Statistical Manual of Mental Disorders – Fourth edition (DSM-IV) | **Both tested polymorphisms of NRG1 are associated with PTSD occurrence**  ***rs35753505***   - Having a CC genotype of rs35753505 led to an increased risk of PTSD among patients   - TT: Reference   - TC: OR: 0.620; 95% CI: 0.329 – 1.189; *p* = 0.176   - CC: OR: 2.077; 95% CI: 1.066 – 4.141; *p* = 0.041   ***rs3924999***   - Having a GG genotype of rs3924999 led to an increased risk of PTSD among patients   - AA: Reference   - AG: OR: 0.492; 95% CI: 0.250 – 1.028; *p* = 0.066   - GG: OR: 2.126; 95% CI: 1.045 – 4.222; *p* = 0.040 |
| Miaskowski et al., 2016 (46); United States | Breast cancer patients having completed surgery  N = 398  Age: 57.5 ± 11.5 years (patients with low anxiety) and 53.4 ± 11.3 years (patients with high anxiety)  Subjects recruited at a local breast cancer centre, 2 public hospitals and 4 community practices | ***Tumour necrosis factor-alpha (TNF-α)***  rs1799964  (The T>C polymorphism)  (Genotypes: TT, TC, CC)  rs3093662  (The A>G polymorphism)  (Genotypes: AA, AG, GG) | - Anxiety | The Spielberger State-Trait Anxiety Inventories (STAI-T, STAI-S) | **The rs1799964 and rs3093662 polymorphisms of the TNF-α gene are associated with anxiety levels**  ***rs1799964***   - Having the CC genotype (i.e. having two C alleles) is associated with a decreased odds of belonging to the high anxiety level class (i.e. having a STAI-T and STAI-S score of ≥32.2 and ≥31.8 respectively)   - OR: 0.12; 95% CI: 0.030 – 0.471; *p* = 0.002   ***rs3093662***   - Having the AG or GG genotypes (i.e. having at least one copy of the minor allele) is associated with an increased odds of belonging to the high anxiety level class (i.e. having a STAI-T and STAI-S score of ≥32.2 and ≥31.8 respectively)   - OR: 4.04; 95% CI: 1.694 – 9.623; *p* = 0.002 |
| Reyes-Gibby et al., 2013 (47); United States | Lung cancer patients who were newly diagnosed (had not undergone cancer treatment)  N = 599  Age: 61 ± 12 years  Subjects were drawn from a previous case-control study. Subjects in that study was recruited at a local cancer centre | ***Interleukin-8 (IL-8)***  IL-8 -251 T>A polymorphism  (rs number not reported)  (Genotypes: TT, TA, AA)  ***Interleukin-1 alpha (IL-1A)***  IL-1A -889 C>T polymorphism  (rs number not reported)  (Genotypes: CC, CT, TT)  ***Interleukin-10 receptor, beta subunit (IL-10RB)***  IL-10RB Lys47Glu polymorphism  (rs number not reported)  (Genotypes: Lys/Lys, Lys/Glu, Glu/Glu)  **Tumor necrosis factor-beta (TNF-β)**  TNF-β Arg13Cys polymorphism  (rs number not reported)  (Genotypes: Arg/Arg, Arg/Cys, Cys/Cys) | - Depression | Items in the 12-Item Short Form Survey (SF-12) | **Only the IL-8 -251 T>A polymorphism is associated with the risk of severe depression. No association was observed for the other three assessed gene polymorphisms**  ***IL-8 -251 T>A polymorphism***   - Carriers of the A allele (patients having either AA or TA genotype) were at lower risk of severe depression, and the difference was marginally significant.   - OR: 0.37; 95% CI: 0.14 – 1.00; *p* = 0.049   ***IL-1A -889 C>T polymorphism***   - This gene polymorphism was not associated with the risk of having severe depression among patients   - OR: 0.30; 95% CI: 0.06 – 1.57; *p* = 0.153   ***IL-10RB Lys47Glu polymorphism***   - This gene polymorphism was not associated with the risk of having severe depression among patients   - OR: 2.58; 95% CI: 0.65 – 10.26; *p* = 0.177   ***TNF-β Arg13Cys polymorphism***   - This gene polymorphism was not associated with the risk of having severe depression among patients   - OR: 1.61; 95% CI: 0.35 –7.40; *p* = 0.541 |
| Saad et al., 2014 (48); United States | Breast cancer patients having completed  N = 398  Age of the overall sample of subjects was not reported.  Subjects recruited at a local breast cancer centre, 2 public hospitals and 4 community practices | ***Interferon-γ receptor 1 (IFNGR1)***  The G>A polymorphism  (rs9376268)  (Genotypes: GG, GA, AA)  ***Interleukin-6 (IL-6)***  The C>G polymorphism  (rs2069840)  (Genotypes: CC, CG, GG)  ***Tumour necrosis factor-α (TNF-α)***  The T>C polymorphism  (rs1799964)  (Genotypes: TT, TC, CC) | - Depression | Centre for Epidemiologic Studies Depression scale  (CES-D) | **The rs9376268 polymorphism of IFNGR1 was associated with a higher severity of depression**   - Compared to GG homozygotes, carriers of the A allele in this polymorphism (i.e. patients with AA or GA genotype) were at higher odds of being in subsyndromal class of depression (with a higher depression severity)   - OR: 1.87; 95% CI: 1.097 – 3.201; *p* = 0.022   **The rs2069840 polymorphism of IL-6 was associated with a higher severity of depression**   - Patients with the homozygous GG genotypes were at higher odds of being in subsyndromal class of depression (with a higher depression severity), compared to those having the CC or CG genotype   - OR: 3.06; 95% CI: 1.165 – 8.054; *p* = 0.023   **The rs1799964 polymorphism of TNF-α was associated with a lower severity of depression**   - Patients with the homozygous CC genotypes were at lower odds of being in subsyndromal class of depression (with a higher depression severity), compared to those having the TT or TC genotype   - OR: 0.13; 95% CI: 0.026 – 0.635; *p* = 0.012 |
| Schillani et al., 2012 (62); Italy | Early breast cancer patients after undergoing surgery and before adjuvant treatments  N = 48 (baseline [T0])  N = 48 (1^st^ follow-up at 1 month [T1])  N = 35 (2^nd^ follow-up at 3 months [T2])  Age: 60.2 ± 1.3 years  Subjects recruited at a local social oncology centre | ***Serotonin-transporter-linked polymorphic region (5-HTTLPR) gene***  Deletion polymorphism in the polymorphic region of serotonin transporter gene leading to a shorter version of the gene  (rs number not reported)  (Genotypes: SS, SL, LL) | - Depression - Anxiety - Anxious preoccupation | Hospital  Anxiety and Depression Scale (HADS)  Mini Mental Adjustment to Cancer Scale (Mini-  MAC) | **The 5-HTTLPR gene polymorphisms were not associated with depression nor anxiety**  The extent of change in depression and anxiety scores between carriers of the S allele and patients having the LL genotypes between T0 and T2 was not significantly different.  ***HADS scores for depression (p = 0.429)***  ***SS and SL (S allele carriers with reduced serotonin transporter expression)***   - T0: 4.31 ± 0.60 - T1: 3.14 ± 0.51 - T2: 3.32 ± 0.77   ***LL (with normal serotonin transporter expression)***   - T0: 3.89 ± 0.75 - T1: 3.05 ± 0.59 - T2: 2.56 ± 0.62   ***HADS scores for anxiety (p = 0.170)***  ***SS and SL (S allele carriers with reduced serotonin transporter expression)***   - T0: 5.41 ± 0.83 - T1: 3.90 ± 0.60 - T2: 4.21 ± 0.97   ***LL (with normal serotonin transporter expression)***   - T0: 3.26 ± 0.69 - T1: 3.16 ± 0.85 - T2: 2.38 ± 0.69   **The 5-HTTLPR gene polymorphisms were associated with scores of anxious preoccupation** The level of anxious preoccupation of patients (patients expressing anxiety as a coping strategy to the experience of the disease) decreased with time. The extent of this decrease was significantly smaller among S allele carriers (SS and SL) compared to LL homozygotes.  ***Mini-MAC scores for anxious preoccupation (p = 0.023)***  ***SS and SL (S allele carriers with reduced serotonin transporter expression)***   - T0: 16.31 ± 0.96 - T1: 13.72 ± 0.86 - T2: 14.21 ± 0.96   ***LL (with normal serotonin transporter expression)***   - T0: 14.79 ± 0.99 - T1: 13.16 ± 1.00 - T2: 10.50 ± 0.66 |
| Sharpley et al., 2018 (65); Australia | Prostate cancer patients, treatment stage not reported  N = 93  Age: 67.9 ± 6.4 years  Subjects recruited from local cancer treatment centres | ***Brain-derived neurotrophic factor (BDNF)***  The Val66Met polymorphism –BDNF 196 G>A  (rs6265)  (Genotypes: Val/Val, Val/Met, Met/Met) | - Depression - Anxiety | Patient health questionnaire-9 scale (PHQ9)  Generalised anxiety disorder-7 item scale (GAD7)  Salivary cortisol levels | **The Val66Met polymorphism had no effect on depression and anxiety of patients**  No significant difference was observed in the depression and anxiety scores between Met allele carriers (Val/Met and Met/Met) and patients bearing the Val/Val genotype  ***Depression scores (p = 0.806)***   - Val/Met and Met/Met: 12.58 ± 2.85 - Val/Val: 12.72 ± 2.41 - F value: 0.061   ***Anxiety scores (p = 0.988)***   - Val/Met and Met/Met: 15.69 ± 5.02 - Val/Val: 15.68 ± 3.58 - F value: 0.000 |
| Suppli et al., 2015 (64); Denmark | Colorectal cancer patients, treatment status not reported  N = 806 (biallelic genotype of 5-HTTLPR determined)  N = 793 (triallelic genotype of 5-HTTLPR determined)  Mean age: 66.4 years  Subjects were the participants of a previous cohort study who were diagnosed to have their colorectal cancer diagnosed between 1998 and 2009 | ***Serotonin-transporter-linked polymorphic region (5-HTTLPR) gene***  Deletion polymorphism in the polymorphic region of serotonin transporter gene leading to a shorter version of the gene  (rs number not reported)  (Genotypes: SS, SL, LL)  The deletion polymorphism with rs25531 polymorphism (The triallelic system)  (The A>G polymorphism in the L allele)  Genotypes grouped based on 5HTTLPR transcriptional activity:   - L_A_L_A_ (high activity); - L_A_L_G_, SL_A_ (medium activity); - L_G_L_G_, SL_G_, SS (low activity) | - Depression | Use of antidepressants | **The 5-HTTLPR deletion polymorphism is not associated with use of antidepressants among patients**  ***Use of antidepressants***   - LL: Reference - SL: HR: 0.92; 95% CI: 0.70 – 1.30; *p* > 0.05 - SS: HR: 1.03; 95% CI: 0.70 – 1.60; *p* > 0.05   **The deletion polymorphism and rs25531 polymorphism is not associated with use of antidepressants among patients**  ***Use of antidepressants***   - L_A_L_A_: Reference - L_A_L_G_, SL_A_: HR: 0.98; 95% CI: 0.70 – 1.40; *p* > 0.05 - L_G_L_G_, SL_G_, SS: HR: 1.08; 95% CI: 0.70 – 1.60; *p* > 0.05 |
| Suppli et al., 2017 (38); Denmark | Patients diagnosed with primary cancer in the colon or rectum, pancreas, lung, breast, prostate, corpus uteri, ovaries, or urinary bladder. Treatment stage not specified  N = 7,320  Mean age: 68.3 years  Subjects were the participants of a previous cohort study who were diagnosed to have their first primary cancer between 1998 and 2013 | ***Serotonin-transporter-linked polymorphic region (5-HTTLPR) gene***  Deletion polymorphism in the polymorphic region of serotonin transporter gene leading to a shorter version of the gene  (rs number not reported)  (Genotypes: SS, SL, LL)  The deletion polymorphism with rs25531 polymorphism (The triallelic system)  (The A>G polymorphism in the L allele)  Genotypes grouped based on 5HTTLPR transcriptional activity:   - L_A_L_A_ (high activity); - L_A_L_G_, SL_A_ (medium activity); - L_G_L_G_, SL_G_, SS (low activity)   ***Brain-derived neurotrophic factor (BDNF)***  The Val66Met polymorphism –BDNF 196 G>A  (rs6265)  (Genotypes: Val/Val, Val/Met, Met/Met)  ***Serotonin 1a receptor (HTR1a)***  The C>G polymorphism  (rs6295)  (Genotypes: CC, CG, GG)  ***Catechol-O-methyl transferase (COMT)***  The G>A polymorphism  (rs4680)  (Genotypes: GG, GA, AA)  ***FK506-binding protein 5 (FKBP5)***  The C>T polymorphism  (rs1360780)  (Genotypes: CC, CT, TT) | - Depression | Use of antidepressants  Hospital contact for depression | **All the assessed gene polymorphisms were not associated with depression, as measured by the use of antidepressants and hospital contact for depression among patients**  ***Use of antidepressants***  ***5-HTTLPR deletion polymorphism***   - LL: Reference - SL: HR: 0.99; 95% CI: 0.84 – 1.15; *p* = 0.80 - SS: HR: 1.07; 95% CI: 0.88 – 1.31; *p* = 0.33   ***5-HTTLPR deletion polymorphism with the rs25531 polymorphism***   - L_A_L_A_: Reference - L_A_L_G_, SL_A_: HR: 0.95; 95% CI: 0.80 – 1.12; *p* = 0.33 - L_G_L_G_, SL_G_, SS: HR: 1.02; 95% CI: 0.85 – 1.24; *p* = 0.73   ***The Val66Met polymorphism of BDNF***   - GG: Reference - GA: HR: 1.03; 95% CI: 0.88 – 1.19; *p* = 0.62 - AA: HR: 1.01; 95% CI: 0.72 – 1.42; *p* = 0.93   ***The C>G polymorphism of HTR1α***   - CC: Reference - CG: HR: 0.97; 95% CI: 0.82 – 1.15; *p* = 0.57 - GG: HR: 1.05; 95% CI: 0.86 – 1.27; *p* = 0.49   ***The G>A polymorphism of COMT***   - GG: Reference - GA: HR: 0.99; 95% CI: 0.82 – 1.19; *p* = 0.83 - AA: HR: 1.06; 95% CI: 0.87 – 1.30; *p* = 0.40   ***The C>T polymorphism of FKBP5***   - CC: Reference - CT: HR: 1.04; 95% CI: 0.89 – 1.21; *p* = 0.53 - TT: HR: 1.07; 95% CI: 0.82 – 1.39; *p* = 0.49   ***Hospital contact for depression***  ***5-HTTLPR deletion polymorphism***   - LL: Reference - SL: HR: 1.04; 95% CI: 0.54 – 2.04; *p* = 0.85 - SS: HR: 1.05; 95% CI: 0.44 – 2.50; *p* = 0.87   ***5-HTTLPR deletion polymorphism with the rs25531 polymorphism***   - L_A_L_A_: Reference - L_A_L_G_, SL_A_: HR: 0.91; 95% CI: 0.45 – 1.82; *p* = 0.69 - L_G_L_G_, SL_G_, SS: HR: 0.80; 95% CI: 0.34 – 1.87; *p* = 0.45   ***The Val66Met polymorphism of BDNF***   - GG: Reference - GA: HR: 1.16; 95% CI: 0.62 – 2.16; *p* = 0.50 - AA: HR: 0.82; 95% CI: 0.15 – 4.48; *p* = 0.74   ***The C>G polymorphism of HTR1α***   - CC: Reference - CG: HR: 1.01; 95% CI: 0.48 – 2.15; *p* = 0.90 - GG: HR: 1.35; 95% CI: 0.60 – 3.07; *p* = 0.29   ***The G>A polymorphism of COMT***   - GG: Reference - GA: HR: 0.73; 95% CI: 0.34 – 1.86; *p* = 0.22 - AA: HR: 0.82; 95% CI: 0.36 – 1.54; *p* = 0.49   ***The C>T polymorphism of FKBP5***   - CC: Reference - CT: HR: 1.29; 95% CI: 0.67 – 2.46; *p* = 0.27 - TT: HR: 1.08; 95% CI: 0.33 – 3.50; *p* = 0.85 |
| Wang et al., 2019 (49); United States | Breast cancer patients having completed surgery, but before the initiation of systemic adjuvant therapy  N = 80  Age: 59.8 ± 5.8 years  Subjects recruited at the cancer institute and cancer centres of a local university | ***Serotonin-transporter-linked polymorphic region (5-HTTLPR) gene***  Deletion polymorphism in the polymorphic region of serotonin transporter gene leading to a shorter version of the gene  (rs number not reported)  (Genotypes: SS, SL, LL) | - Depression | Beck Depression Inventory (BDI-II) | **Among subjects with breast cancer, the deletion polymorphism had no significant association with depression**  No significant difference was observed between the BDI-II score of the short allele carriers (SS or SL – genotypes with lower expression of serotonin transporter) and that of LL homozygotes (normal serotonin transporter expression), at baseline (at enrollment, T1 (6 months after enrollment) and T2 (12 months after enrollment).  ***At baseline***   - SS and SL: 4.92 ± 0.80 - LL: 6.44 ± 1.12 - *p* = 0.270   ***At T1***   - SS and SL: 6.89 ± 0.80 - LL: 6.15 ± 1.12 - *p* = 0.591   ***At T2***   - SS and SL: 5.90 ± 0.81 - LL: 6.27 ± 1.14 - *p* = 0.790   Nevertheless, the increase in depression severity among the patients not having the LL genotype (i.e. SL and SS) was significant between baseline and T1 (*p* = 0.002) |
| Young et al., 2017 (50); United States | Breast cancer patients undergoing chemotherapy  N = 51  Age: 51.9 ± 11.4 years  Secondary analysis of data from a previous randomised controlled trial, where participants were recruited at a local cancer centre | ***Catechol-O-methyl transferase (COMT)***  The G>A polymorphism  (rs4680)  (Genotypes: GG, GA, AA) | - Anxiety | Hospital Anxiety and Depression Scale (HADS) | **The rs4680 polymorphism of COMT is associated with anxiety severity among patients at diagnosis, but this association was no longer occurred after surgery and chemotherapy**   - Patients bearing the AA genotype had higher level of anxiety compared to those having the GG or GA genotype (*p* = 0.005; numerical data on anxiety score were not explicitly reported) - 11.3% of the change in anxiety score was accounted by the difference in genotypes of patients |
| Zerbinati et al., 2021 (63); Italy | Breast cancer patients. Treatment stage not reported  N = 145  Age: 55.9 ± 9.0 years  Subjects recruited at day-hospital clinics of a local university hospital | ***Serotonin-transporter-linked polymorphic region (5-HTTLPR) gene***  Deletion polymorphism in the polymorphic region of serotonin transporter gene leading to a shorter version of the gene  (rs number not reported)  (Genotypes: SS, SL, LL) | - Posttraumatic stress | Impact of Event Scale (IES) | **The 5-HTTLPR gene polymorphisms were associated with the risk of posttraumatic stress**  Patients bearing the LL genotype were at higher risk of having posttraumatic stress symptoms (based on the IES score) as a result of having cancer-related problems compared to those having the SL and SS genotype.  ***Effect of cancer-related problems among patients of various genotype***  LL – Effect: 2.43; 95% CI: 0.62 – 4.23; p < 0.01  SL – Effect: -0.57; 95% CI: -2.04 – 0.89; p = 0.43  SS – Effect: -0.72; 95% CI: -2.56 – 1.11; p = 0.44  Overall, 5-HTTLPR gene polymorphism had a significant moderating effect of the effect of cancer-related problems on IES score (a measure of posttraumatic stress), explaining 5% of the variance of IES score (*p* = 0.02) |

Abbreviations: AOR: adjusted odds ratio; HR: hazard ratio; OR: odds ratio; CI: confidence interval
